# Layer by Layer: Assessing AI Diagnostic Accuracy With Incremental Case Information in Neuroradiology

**DOI:** 10.7759/cureus.85874

**Published:** 2025-06-12

**Authors:** Golnaz Lotfian, Miral Jhaveri, Sumeet G Dua, Pokhraj P Suthar

**Affiliations:** 1 Department of Diagnostic Radiology and Nuclear Medicine, Rush University Medical Center, Chicago, USA

**Keywords:** ai, gemini, ml, neuroradiology, nlp

## Abstract

Aim

Artificial intelligence (AI) has proven tremendous potential in improving diagnostic accuracy and efficiency in radiology. This study assesses the diagnostic performance of Google Gemini (version 1.5 Flash; Google DeepMind, Mountain View, California, USA), a proprietary large language model, in interpreting challenging diagnostic cases from the *American Journal of Neuroradiology’s (AJNR)* "Case of the Month" series.

Materials and methods

We analyzed 143 neuroradiology cases spanning brain, head and neck, and spine areas. Each case evolved over four weeks, starting with clinical history and followed by incremental imaging findings. Google Gemini was often prompted with the question, "What is the diagnosis?" Its accuracy was assessed at each level and across specialty categories. The data used were publicly available, and no ethical approval was necessary.

Results

Gemini's diagnosis accuracy improved with new case data, from 3.5% with history alone to 45.7% after complete imaging was supplied. Accuracy by category was highest in spine cases (51.9%), followed by head and neck (45.5%) and brain (44.0%). A chi-square test for trend verified that the performance increase over time was statistically significant (p < 0.0000000001).

Conclusion

Google Gemini displays moderate diagnosis accuracy that improves with accumulated information. While encouraging, its shortcomings underline the necessity for continual validation and transparency. This study shows the expanding relevance of AI in neuroradiology and the necessity of comprehensive evaluation before clinical integration.

## Introduction

Artificial intelligence (AI) and natural language processing (NLP) are progressively influencing the future of radiology, with AI-driven solutions enhancing diagnostic precision, optimizing workflows, and aiding physicians in interpreting complex imaging data [1]. The capacity of AI to transform clinical decision-making is exemplified by systems like Google Gemini (Google DeepMind, Mountain View, California, USA), which are engineered to evaluate and synthesize large volumes of multimodal information [2,3]. As these advanced tools become more integrated into diagnostic environments, it is essential to assess not only their peak accuracy but also their performance under real-world constraints, such as fragmented or stepwise clinical data presentation.

This study evaluates the diagnostic performance of Google Gemini using 143 neuroradiology cases from the *American Journal of Neuroradiology’s (AJNR)* "Case of the Month" series. To simulate realistic diagnostic progression, the model was prompted incrementally, receiving only newly released clinical and imaging information at each weekly stage. This design mimics how radiologists often refine differential diagnoses as new data becomes available. The primary objective of this study is to determine how well a large language model (LLM) adapts to this staged, iterative information flow and whether it can produce accurate diagnostic assessments without retrospective access to prior data.

Beyond Gemini, domain-specific AI tools like Zebra Medical Vision (Nanox Imaging Ltd., Neve Ilan, Israel) and Aidoc (Aidoc Medical Ltd., Tel Aviv, Israel) have demonstrated high accuracy in applications such as hemorrhage and pulmonary embolism (PE) detection [4,5]. Nevertheless, significant challenges persist, chief among them the generalizability of models across institutions, the opacity of proprietary systems, and the need for interpretability in clinical decision-making [6,7]. Predictive modeling, another frontier in radiologic AI, continues to show promise in managing stroke, traumatic brain injury, and intracranial hemorrhage (ICH) [8]. This paper seeks to position the staged-evaluation framework as a new lens through which AI performance can be stress-tested under clinically plausible conditions, adding nuance to the ongoing conversation around medical AI’s capabilities and limitations.

## Materials and methods

This study evaluated the diagnostic accuracy of Google Gemini (version 1.5 Flash), a commercially available LLM, using clinical case materials from the AJNR publicly accessible “Case of the Month” series. The model was assessed on its ability to produce correct diagnoses when presented with clinical and imaging information revealed incrementally across a four-week case structure. The study adhered to the STARD (Standards for Reporting Diagnostic Accuracy Studies) framework. Since all data sources were publicly available, no institutional review board approval was required.

A total of 144 AJNR cases published between November 2011 and October 2023 were screened. One case (February 2018) was excluded due to incomplete imaging data, resulting in 143 cases for final analysis. Each case typically followed a four-week release cycle: Week 1: clinical history, and Weeks 2-4: stepwise release of imaging findings and supplementary clinical details.

In cases that included pathology slides, those were reviewed to preserve diagnostic context, though any figure captions disclosing final diagnoses were excluded from input to avoid bias.

To simulate real-world diagnostic reasoning, Gemini was prompted at each weekly stage only with the newly released information, omitting prior weeks’ content. This design was intended to replicate how clinicians often update their differential diagnoses as new data becomes available, rather than synthesizing all information retrospectively. At each stage, the prompt used was identical: “Given the above information, what is the most likely diagnosis?”

The interaction was conducted in a zero-shot setting via Gemini’s commercial web-based interface (version 1.5 Flash, March 2024 release), with no fine-tuning, additional context, or prompt engineering beyond the weekly case text. Because Gemini cannot interpret images directly, textual summaries of imaging findings were curated from the AJNR case material. These summaries were standardized across all cases to ensure a consistent description of findings. No image content was altered or paraphrased beyond maintaining fidelity to AJNR’s original weekly narrative structure. To ensure accuracy and clinical relevance, all case summaries and corresponding input text were independently reviewed and validated by three board-certified, fellowship-trained neuroradiologists with subspecialty expertise in brain, spine, and head and neck imaging.

Gemini’s output at each stage was independently reviewed by the same three neuroradiologists. A response was judged as “correct” only if it matched or appropriately captured the final AJNR diagnosis (based on their published solution) either directly or via clinically synonymous terminology. Any discrepancies or borderline calls were resolved by consensus. Performance was tracked across four data stages (Weeks 1-4), allowing assessment of information-dependent diagnostic improvement.

Cases were categorized into three neuroradiology domains: Brain (n = X), Head and Neck (n = Y), Spine (n = Z). This stratification enabled subgroup analysis to assess whether AI diagnostic performance varied by anatomic region, which may reflect inherent strengths or limitations of current LLMs in different imaging contexts.

All statistical analyses were conducted in Microsoft Excel (Office 365; Microsoft® Corp., Redmond, WA, USA). Diagnostic accuracy was computed as a proportion at each weekly stage. A chi-square test for trend was performed to assess whether accuracy improved across the four stages in a statistically significant manner (p < 0.05 considered significant).

**Figure 1:**
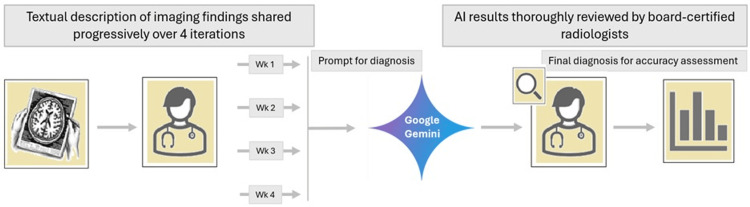
Data processing and assessment workflow Image credit: Golnaz Lotfian, the author.

A visual schematic summarizing the diagnostic pipeline, prompting procedure, and evaluation process is provided in Figure 1.

## Results

The diagnostic efficacy of Google Gemini fluctuated based on the volume of information supplied. When solely the patient history was provided, Google Gemini attained a diagnosis accuracy of 3.5%. Incorporating imaging findings from the initial week enhanced the accuracy to 10.5%. Integrating patient history and imaging data from the initial two weeks increased accuracy to 18.4%. Incorporating imaging findings from the third week yielded a diagnosis accuracy of 29.6%. Ultimately, upon receiving the complete patient history and imaging results from the four-week period, Google Gemini attained a diagnosis accuracy of 45.7%.

**Figure 2:**
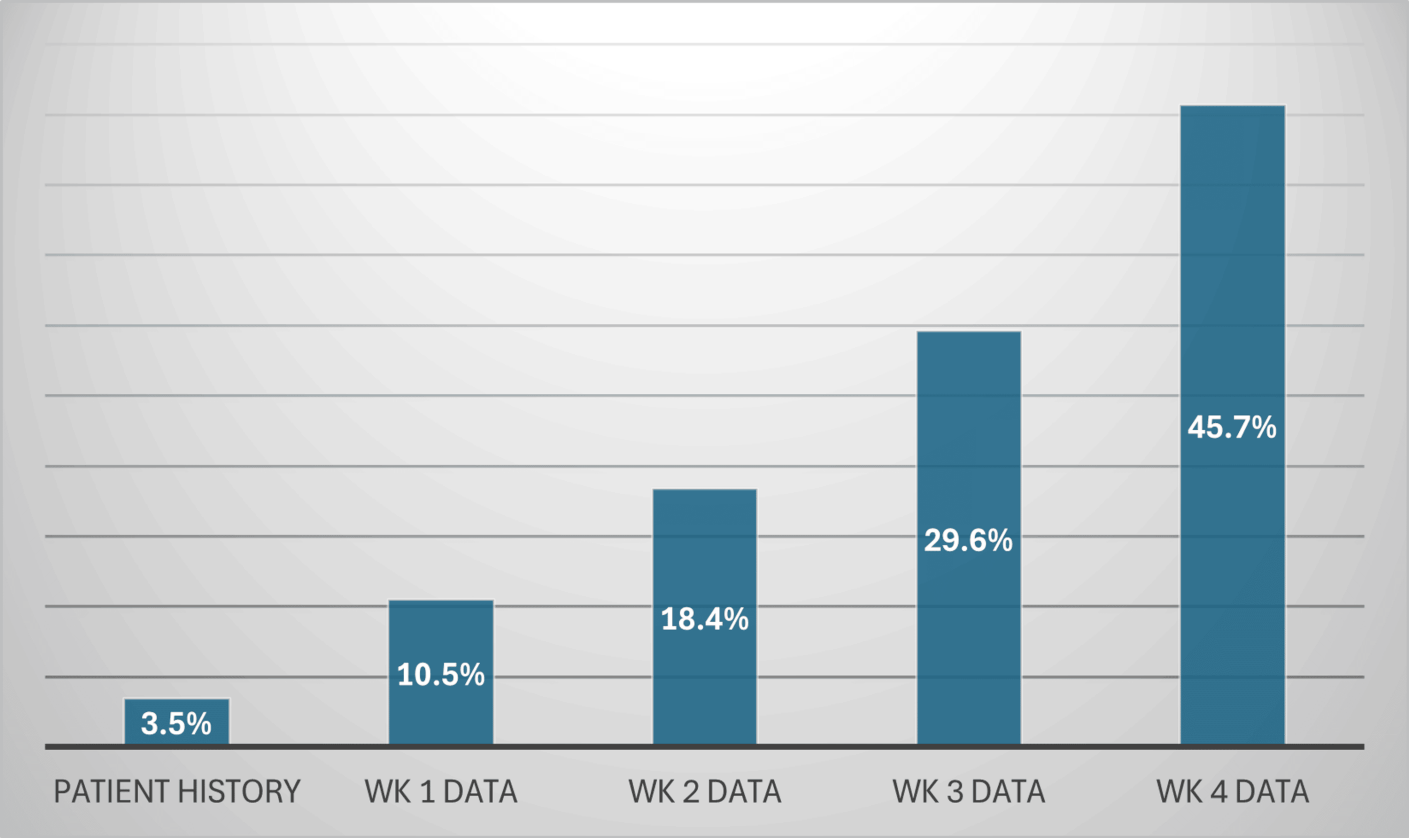
Gemini’s performance with increased availability of diagnostic data

**Figure 3:**
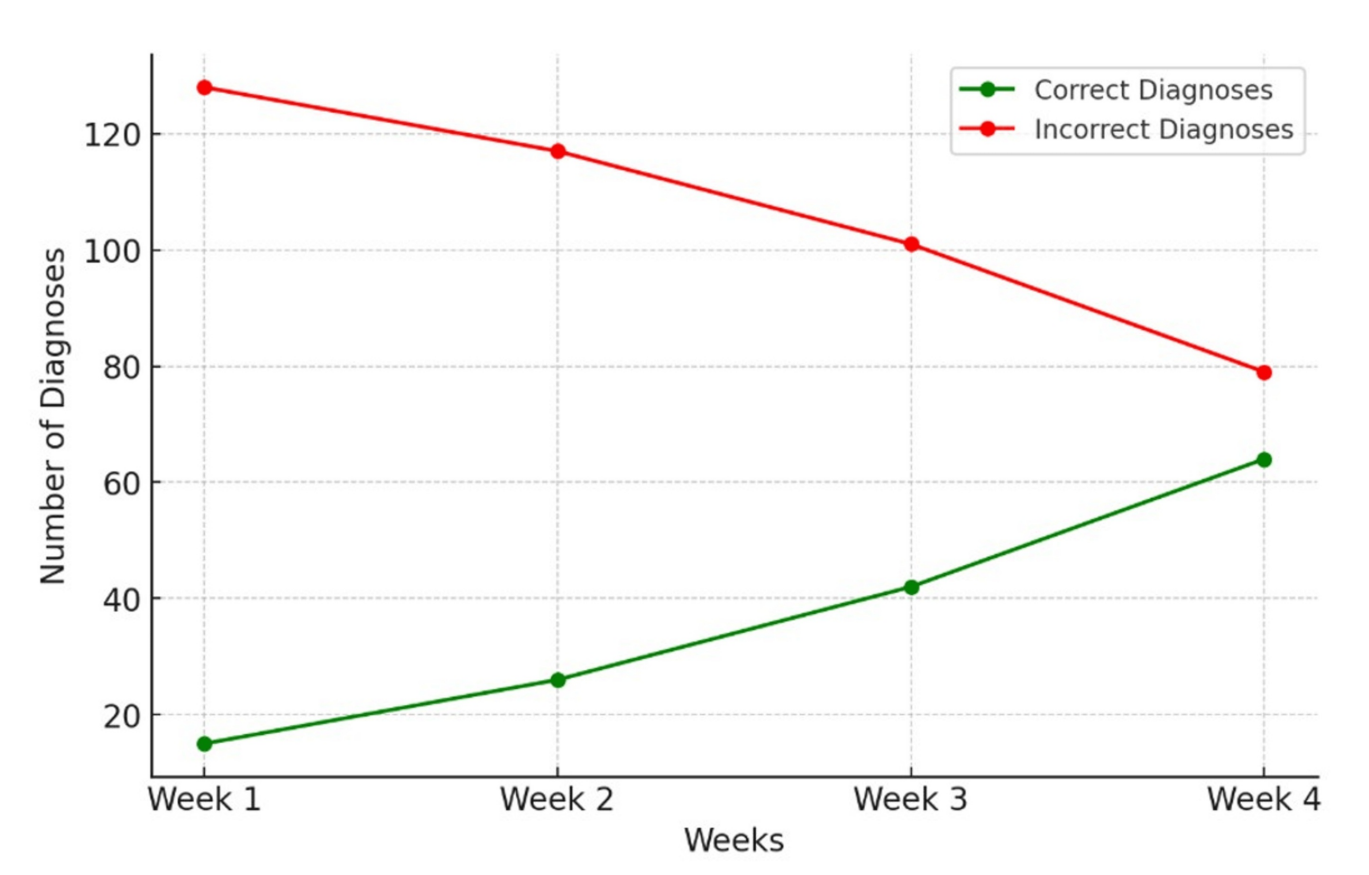
Trend of Gemini’s correct vs. incorrect responses over time

A chi-square test for trend was conducted to assess whether the observed improvement in diagnostic accuracy over time was statistically significant. The test yielded a chi-square statistic of 50.52 with a corresponding p-value of 6.2 × 10^-11^, indicating that the increasing accuracy trend is highly significant and unlikely due to chance. This statistical confirmation reinforces the observation that Gemini's diagnostic precision improves as additional information is supplied. The accuracy metrics and trend analysis are displayed in Figures 2-3.

**Figure 4:**
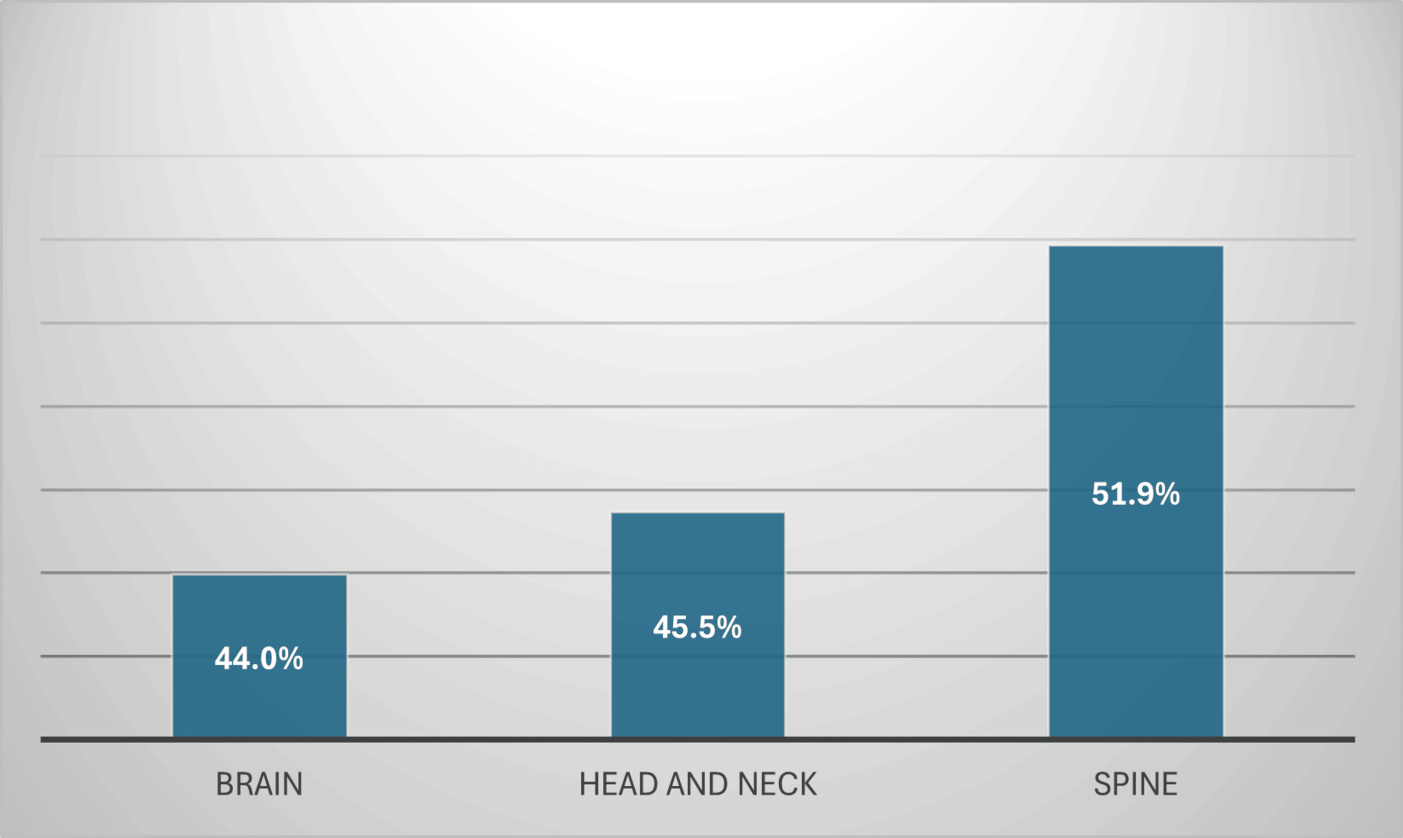
Gemini’s performance across the three main neuroradiology categories

Google Gemini's diagnostic accuracy varied across the categories of brain, head and neck, and spine. In cases pertaining to the brain, it attained an accuracy of 44.0%. The accuracy in the head and neck category was marginally elevated to 45.5%. The highest accuracy was noted in spine cases, with Google Gemini accurately diagnosing 51.9% of the instances (Figure 4).

## Discussion

Integration of NLP and AI in neuroradiology

Imaging Detection and Recognition

NLP and AI techniques are integrated into neuroradiology to improve diagnostic accuracy, efficiency, and clinical decision-making [1-3]. For instance, Aidoc and Zebra Medical Vision represent significant advancements in this field, utilizing deep learning and NLP to examine large volumes of radiology reports and imaging data, thus enhancing the interpretation of unstructured information and providing decision support for healthcare practitioners [4,5].

Aidoc has been utilized in clinical settings for the detection of PE in COVID-19 patients, demonstrating strong performance with a sensitivity of 93.2%, specificity of 99.6%, and an overall accuracy of 98.4% for mild cases. Accuracy further improved for intermediate and severe cases, reaching 96.7% and 97.2%, respectively [3,4]. This example underscores the value of AI in managing complex and rapidly evolving clinical scenarios, such as those seen during the COVID-19 pandemic [3,4]. Similarly, Zebra Medical Vision has applied AI to diagnostic imaging with significant impact, achieving 98% accuracy in detecting hemorrhages on CT scans and reducing unnecessary follow-ups by 70% [5]. These examples illustrate how advanced, readily available AI models can enhance diagnostic precision and streamline clinical workflows across diverse applications.

Despite these developments, the widespread adoption of AI in radiology faces challenges, including regulatory barriers, building clinician trust in AI assessments, and demonstrating the long-term therapeutic efficacy of these tools. To optimize the effectiveness of AI tools in improving clinical outcomes, it is crucial to establish stringent data validation procedures [9,10]. Validating AI models is essential to ensure reliable performance across diverse patient demographics, settings, and clinical scenarios while complying with stringent clinical standards.

Disease Recognition and Diagnosis

AI has transformed neuroradiology by markedly improving the ability to identify and diagnose disorders of the brain, head and neck, and spine. These gains have proven especially significant in domains where nuanced alterations in imaging data may signify disease yet are challenging for human clinicians to identify reliably. AI models, particularly those employing deep learning techniques like convolutional neural networks (CNNs), have demonstrated significant efficacy in automating and enhancing the precision of disease identification across diverse imaging modalities, including CT, MRI, and PET scans.

In the realm of brain imaging, AI methods have been effectively utilized to identify brain malignancies, especially gliomas. Zhuge et al. conducted a study revealing that AI systems trained on MRI data attained a sensitivity of 90% and a specificity of 91% in glioma identification [11]. This performance was analogous to seasoned radiologists, who exhibited an average sensitivity of 89% and specificity of 88% [11]. Such elevated accuracy is essential in clinical environments where early diagnosis and accurate tumor delineation are imperative for therapy planning.

In head and neck imaging, AI has also demonstrated significant efficacy in identifying conditions such as head and neck malignancies. An AI-based tumor segmentation study on multi-parametric MRI scans showed that the tool attained a Dice similarity coefficient (DSC) of 0.85 in segmenting tumors within the oropharyngeal area [12]. The significant enhancement in segmentation accuracy facilitated improved radiation planning and precise targeting of malignant cells, which is crucial for treatment effectiveness and reducing harm to healthy tissues.

In summary, AI has proven its ability to precisely identify and diagnose diseases across multiple areas of neuroradiology, including brain tumors, head and neck cancers, and spinal disorders. These skills boost diagnostic precision and assist in treatment planning, potentially improving patient clinical outcomes.

Predictive Modeling

Predictive modeling has emerged as a pivotal use of AI in neuroradiology. It is supported by an expanding corpus of research demonstrating its capacity to forecast various elements of stroke, brain injury, and other neurological disorders. Through the analysis of imaging data alongside clinical characteristics, predictive models can discern high-risk patients, anticipate treatment results, and inform clinical decision-making. Machine learning methodologies, such as support vector machines (SVM), random forests, and deep learning algorithms, are frequently utilized for predictive tasks in neuroradiology, with AI models exhibiting robust predictive efficacy [8].

An instance of predictive modeling in acute stroke pertains to identifying the hyperdense middle cerebral artery (MCA) dot sign, signifying a thromboembolism in acute MCA infarcts [13]. A recent study utilized machine learning to identify the MCA dot sign on CT scans of individuals suspected of acute ischemic stroke. The model attained a sensitivity of 97.5% (39 out of 40 identified MCA dot signals) with a false-positive rate of 0.5 per image, illustrating its proficiency in correctly predicting thromboembolism in this high-risk patient cohort [13]. This prediction approach assists doctors in swiftly identifying potential stroke patients, facilitating prompt interventions, and minimizing diagnostic delays [13].

Predictive modeling has been employed in malignant hemispheric infarction to automate the assessment of cerebral edema, a vital prognostic indication for patients [14]. Traditionally, evaluating cerebral edema and tracking alterations in cerebrospinal fluid (CSF) volumes by sequential CT scans has been a labor-intensive and time-consuming endeavor. A machine learning model based on random forests, along with geodesic active contour segmentation, was created to automate this procedure. The model demonstrated strong concordance with manual segmentation, with a Pearson correlation coefficient of 0.879 (p < 10^-6^), suggesting that AI can accurately forecast ΔCSF alterations [14]. This forecast is essential for identifying when a midline shift, indicative of significant edema, is probable, facilitating early intervention for patients susceptible to additional neurological decline [14].

Moreover, machine learning models have been utilized to forecast the probability of symptomatic ICH after thrombolysis treatment for acute ischemic stroke [15]. A study employed CT brain scan images from 116 individuals, comprising 16 who manifested symptomatic intracerebral hemorrhage following thrombolysis. The SVM classifier, integrated with clinical severity scores, achieved a predictive capability for symptomatic ICH risk, evidenced by an area under the receiver operating characteristic curve (AUC) of 0.744, surpassing the performance of established prognostic instruments such as the Hemorrhage After Thrombolysis (HAT) score (AUC 0.629) and the SEDAN (Sugar, Early infarct signs, Dense artery sign, Age, and NIH Stroke Scale) score (AUC 0.626) [15]. This illustrates how AI might enhance the precision of risk prediction models, facilitating improved patient stratification for thrombolysis treatment [15].

These examples highlight the significant potential of AI and predictive modeling in enhancing decision-making within therapeutic environments. AI models can improve clinical decision-making by forecasting stroke risks, treatment problems, and patient outcomes, hence potentially enhancing clinical results and optimizing resource allocation within healthcare systems.

Continuous improvement

Data Validation and Model Reliability

Data validation is essential for enhancing the precision of AI and NLP models, especially in intricate domains such as medical diagnoses. AI and machine learning algorithms depend significantly on high-quality, annotated datasets to learn and adjust to real-world situations [16]. This process is fundamental in radiology, where AI models need to handle diverse imaging data, such as MRI, CT scans, and other modalities, to produce reliable results for clinicians [17].

In a study examining multiple sclerosis lesion detection, AI was employed to produce double inversion recovery (DIR) and phase-sensitive inversion recovery (PSIR) images, which are conventionally challenging to acquire in clinical environments [18]. The efficacy of AI-generated images was assessed against MRI-acquired DIR and PSIR images in a multicenter study involving 202 patients and 1154 lesions. The AI model demonstrated enhanced efficacy in identifying lesions on DIR pictures relative to MRI-acquired images, achieving a 35% superior identification rate of lesions [18]. The results highlight the significance of adequate model training and validation by contrasting AI-generated images with clinical reference images. The study confirmed that the AI model's output was dependable and consistent with clinical standards.

This exemplifies the training and validation of AI to enhance diagnostic efficacy and augment the value of AI-generated images. This work, particularly in clinical environments, underscores the necessity of verifying AI models using real-world data to guarantee they fulfill physicians' diagnostic requirements and deliver precise outcomes across diverse patient demographics.

In the context of CNNs employed for tumor segmentation in head and neck cancer, there are many examples of successful training and validation of MRI input channels to attain precise lesion delineation [19]. In one particular study, seven distinct MRI sequences were employed to train the CNN, and the model's performance was evaluated by excluding one MRI channel sequentially. The results indicated that eliminating specific input channels, such as the T2* sequence, adversely affected segmentation accuracy, whilst others, like the apparent diffusion coefficient (ADC) channel, exhibited minimal impact [19]. The optimal model encompassed all MRI sequences, attaining a DSC of 0.65, underscoring the significance of data selection and input validation in the training of AI models for clinical applications [19].

These studies emphasize a critical element of AI and NLP model validation: the comprehension of data input contributions and their effect on performance. In both instances, training AI models on diverse data types, such as MRI sequences for tumor segmentation or AI-generated images for lesion detection, ensures robust generalization across many contexts, enhancing diagnostic accuracy. The significance of data validation is paramount, as it directly affects the model's sensitivity, specificity, and overall diagnostic precision.

In our data validation study using Google Gemini, differences between AI outputs and expert evaluations were found, highlighting the need for continued refinement of such readily available proprietary AI models. This process is crucial for ensuring that models such as Gemini operate consistently and correctly in clinical or research environments. The significance of data validation is further exemplified by contrasting Google Gemini with ChatGPT in a real-world diagnostic validation environment.

A comparison of performance trajectories between Gemini and ChatGPT, using the same dataset and a methodologically consistent process, reveals distinct patterns in diagnostic accuracy over time [20]. Gemini demonstrated a slower start but improved at a markedly accelerated rate as more data were assimilated [20]. This illustrates how a model such as Gemini, which implements structured data validation and feedback mechanisms, can perpetually improve its performance, hence increasing its accuracy in diagnostic functions. ChatGPT, although initially demonstrating superior performance in the initial weeks, shows diminished accuracy enhancements, potentially attributable to variations in its validation techniques or the intricacy of the validation procedure. These distinctions underscore the essential function of data validation in enhancing AI model performance, wherein iterative validation, rectification of inconsistencies, and self-learning propel the model's advancement toward greater accuracy.

Without stringent validation, AI models may fail to realize their promise, resulting in erroneous diagnoses and eroding physician trust [21]. The ongoing feedback from data validation rectifies these deficiencies, enabling the model to adjust to various clinical situations, hence guaranteeing its appropriateness for practical applications.

Additionally, cross-validation is an effective method employed to enhance model correctness. This method involves partitioning the dataset into subsets, wherein each subset is utilized for model testing while the remaining subsets serve for training [22]. This ensures that the model does not overfit to a specific data set and generalizes more effectively to novel data [22]. In neuroradiology AI models, cross-validation is crucial for evaluating the model across several institutions, demographic cohorts, and imaging data sources.

Continuous and thorough data validation is essential for enhancing AI and NLP models. Although first-accuracy outcomes may appear favorable, continuous validation and feedback mechanisms are essential to ascertain that these models can manage real-world unpredictability and uphold a high level of performance. As AI technologies such as Gemini and others are incorporated into clinical practice, the demand for stringent validation to enhance their efficacy will escalate. This validation effort will enable AI models to develop into dependable tools for physicians, hence improving diagnostic accuracy and patient outcomes.

Limitations of Proprietary AI Models

Proprietary AI models, such as those developed by Aidoc, Zebra Medical Vision, and Google Gemini, exhibit potential for improving diagnostic accuracy; nonetheless, their implementation in the medical and healthcare fields is fraught with specific limitations. These limitations can significantly impact patient care, clinical procedures, and the extensive adoption of AI technologies in healthcare [6].

A major concern surrounding proprietary AI models is their lack of transparency [7]. Because these models are developed and maintained by private companies, the fundamental mechanisms - including algorithms, databases, and decision-making processes - are often concealed [7]. This presents challenges for healthcare professionals who depend on the AI's diagnostic outcomes, as they cannot easily assess the rationale behind the AI's decision-making or determine if its reasoning aligns with clinical guidelines or standards of care. Lack of transparency hinders physicians' ability to identify when and how AI may fail, eroding trust and hindering the use of AI solutions [23].

Another significant challenge is the generalization of proprietary AI models to perform properly across varied clinical settings, patient demographics, and healthcare contexts [24]. Proprietary models are often trained on data from certain hospitals or patient demographics, limiting their ability to generalize to different settings or diverse populations. Studies evaluating LLMs in healthcare demonstrated that AI models trained on clinical notes from a specific hospital performed inadequately when tested with data from other hospitals, particularly those with small sample sizes, older patients, and individuals with higher comorbidities [25]. These studies have revealed that limitations associated with generalization have the potential to significantly hinder their effectiveness in hospitals with limited data or different demographics [26]. These limitations in model generalization are especially troubling in healthcare, where patient demographics and disease characteristics may vary considerably. An AI model trained in a specific demography may demonstrate reduced efficacy when utilized on underrepresented populations in the training data, leading to diagnostic biases or inaccurate predictions [27].

Studies have shown multiple fine-tuning methodologies, including local fine-tuning (hospital-specific adjustments), have improved the AI and LLM models' relevance and precision, particularly in data-deficient contexts [28]. This highlights the imperative for ongoing model refinement and adjustment to local healthcare environments to ensure the effectiveness of AI technologies in diverse clinical contexts.

In healthcare, generalization difficulties can result in disparities in care, particularly for marginalized communities, and may compromise the precision and fairness of AI-assisted diagnoses [28]. Proprietary models that fail to generalize successfully may inadvertently reinforce existing biases or neglect essential conditions in diverse patient populations, limiting their broader application and reducing their utility in clinical practice.

**Figure 5:**
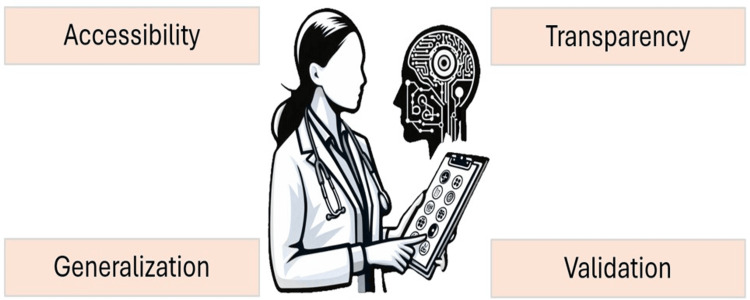
Keys to reliable interaction between human and artificial intelligence data Image credit: Golnaz Lotfian, the author.

To address these challenges, ongoing validation, the use of more extensive datasets, improved transparency, and a stronger focus on accessibility and generalizability are essential in AI model development. These efforts will help ensure that proprietary AI systems are resilient, equitable, and reliable, paving the way for their responsible integration into clinical practice (Figure 5).

Limitations of the Current Study

This research possesses multiple limitations. The AI model was initially evaluated using textual descriptions of imaging findings instead of direct image interpretation, which may not accurately reflect real-world radiologic assessment. Secondly, as a private model, the internal methods and training data of Google Gemini are obscure, hindering interpretability and reproducibility. Third, although the dataset is diverse, it is sourced from a curated academic resource (AJNR “Case of the Month”), which may not accurately represent the range or frequency of pathologies observed in standard clinical practice. The study also failed to evaluate response variability or consistency across several prompts, and human-AI contact was limited to one diagnostic question per stage, which may have underexploited the model's potential. These concerns underscore the necessity for more validation across extensive clinical datasets and more transparent AI systems.

## Conclusions

This study demonstrates that Google Gemini improves diagnostic accuracy with incremental data but still has notable limitations. It reinforces the broader conversation about AI integration in radiology, particularly the importance of transparency, validation, and generalizability.

Predictive modeling shows potential for enhancing clinical decision-making, yet continued improvement in model validation and transparency is critical. As AI progresses, it must be held to high standards of reliability and clinical relevance. Ultimately, AI should complement - not replace - human expertise. Ongoing development and oversight are essential to realizing its full potential in improving patient care.
